# Preoperative Systemic Inflammatory Biomarkers Are Independent Predictors of Disease Recurrence in ER+ HER2- Early Breast Cancer

**DOI:** 10.3389/fonc.2021.773078

**Published:** 2021-11-04

**Authors:** Marta Truffi, Francesca Piccotti, Sara Albasini, Valentina Tibollo, Carlo Francesco Morasso, Federico Sottotetti, Fabio Corsi

**Affiliations:** ^1^ Nanomedicine and Molecular Imaging Lab, Istituti Clinici Scientifici Maugeri IRCCS, Pavia, Italy; ^2^ Breast Unit, Surgery Department, Istituti Clinici Scientifici Maugeri IRCCS, Pavia, Italy; ^3^ Laboratory of Informatics and Systems Engineering for Clinical Research, Istituti Clinici Scientifici Maugeri IRCCS, Pavia, Italy; ^4^ Medical Oncology, Istituti Clinici Scientifici Maugeri IRCCS, Pavia, Italy; ^5^ Department of Biomedical and Clinical Sciences “L. Sacco”, Università di Milano, Milano, Italy

**Keywords:** systemic inflammatory biomarkers, early breast cancer, predictive factors, lymphocyte ratios, disease recurrence

## Abstract

The host’s immune system plays a crucial role in determining the clinical outcome of many cancers, including breast cancer. Peripheral blood neutrophils and lymphocytes counts may be surrogate markers of systemic inflammation and potentially reflect survival outcomes. The aim of the present study is to assess the role of preoperative systemic inflammatory biomarkers to predict local or distant relapse in breast cancer. In particular we investigated ER+ HER2- early breast cancer, considering its challenging risk stratification. A total of 1,763 breast cancer patients treated at tertiary referral Breast Unit were reviewed. Neutrophil-to-lymphocyte (NLR), platelet-to-lymphocyte (PLR) and lymphocyte-to-monocyte (LMR) ratios were assessed from the preoperative blood counts. Multivariate analyses for 5-years locoregional recurrence-free (LRRFS), distant metastases-free (DMFS) and disease-free survivals (DFS) were performed, taking into account both blood inflammatory biomarkers and clinical-pathological variables. Low NLR and high LMR were independent predictors of longer LRRFS, DMFS and DFS, and low PLR was predictive of better LRRFS and DMFS in the study population. In 999 ER+ HER2- early breast cancers, high PLR was predictive of worse LRRFS (HR 0.42, p=0.009), while high LMR was predictive of improved LRRFS (HR 2.20, p=0.02) and DFS (HR 2.10, p=0.01). NLR was not an independent factor of 5-years survival in this patients’ subset. Inflammatory blood biomarkers and current clinical assessment of the disease were not in agreement in terms of estimate of relapse risk (K-Cohen from -0.03 to 0.02). In conclusion, preoperative lymphocyte ratios, in particular PLR and LMR, showed prognostic relevance in ER+ HER2- early breast cancer. Therefore, they may be used in risk stratification and therapy escalation/de-escalation in patients with this type of tumor.

## Introduction

Escalation and de-escalation of treatments is of paramount importance in early breast cancer (1). However, prediction of local or distant failure risk is needed to achieve a personalized medicine. Traditional clinical and pathological features (i.e. nodal status, Ki67%, grading, etc.) are not always able to actually predict disease relapse, especially in ER+ early breast cancer (2, 3). For this reason to predict the risk and address proper treatments can be challenging. Genomic assays such as EndoPredict or OncoType DX are expensive, not widely available and their role in clinical practice is still controversial (4).

In the last decades the relevance of the host’s immune system has been highlighted as crucial in determining clinical outcomes in many cancers, including breast cancer (5, 6). The host immune response has shown a remarkable impact on cancer progression (7). In particular the density and spatial localization of CD8+ infiltrate within central core and invasive margins of tumor (evaluated by the Immunescore) are becoming important prognostic predictors, playing a role in the balance between tumor immune surveillance and escape (8). Tumor-infiltrating lymphocytes (TILs) support antitumor cytotoxic response and are favorable prognostic features along with low densities of immunosuppressive elements like neutrophils and myeloid-derived suppressor cells (9, 10).

Because of their accessibility, peripheral blood neutrophils and lymphocytes counts have gained a broad interest in cancer prognostication as surrogate markers of inflammation and immune response. Easily-gettable and affordable blood-derived inflammatory biomarkers, such as the neutrophil-to-lymphocyte ratio (NLR), have recently demonstrated that the status of immunity often reflects survival outcomes (11). Some evidences suggested the role of these ratios in breast cancer too. In a recent meta-analysis, it was found that NLR has a significant prognostic effect on the overall and disease-free survival rates, suggesting that it could be a promising prognostic marker (12). Platelet-to-lymphocyte ratio (PLR) and lymphocyte-to-monocyte ratio (LMR) are less frequently studied, but they may also be prognostically informative in breast cancer (13–15).

Despite such evidences, results remain discordant, probably due to the study design. Some studies focus on a single molecular subtype or evaluate preoperative blood-derived lymphocyte ratios in presence of specific clinical-pathological characteristics or settings (16). Furthermore, the follow-up period considered in the analyses is often relatively short. This observation is crucial when considering that luminal breast cancers carry a consistent long-term risk of recurrence (17). Finally, in the last years many studies focused on the predictive role of inflammatory biomarkers in breast cancer patients treated by neoadjuvant chemotherapy and generally affected by a specific molecular subtype breast cancer (18, 19). Therefore previous studies investigated small series and highly selected cohorts of patients with breast cancer, while there is a lack of large unselected cohorts of early breast cancer patients.

In the present study we assessed the role of preoperative blood-derived lymphocyte ratios (NLR, PLR, LMR) to predict local or distant relapse in 1,763 breast cancer patients reviewed retrospectively. In particular, the prognostic relevance of lymphocyte ratios was investigated in ER+ HER2- early breast cancers where risk stratification is more challenging.

## Materials and Methods

### Patient Selection

Patients included in this study were retrospectively collected from the prospective database of the EUSOMA-accredited Breast Unit of Istituti Clinici Scientifici Maugeri (Pavia, Italy). Inclusion criteria were: proven diagnosis of invasive breast cancer; candidate to upfront breast surgery; age >18 years. Patients with benign lesions and patients undergoing a neoadjuvant chemotherapy were excluded from the study. Patients received adjuvant treatments (radiotherapy, chemotherapy, biological therapy, hormonal therapy) according to the standard of care. Data were obtained from a study protocol authorized by the Institutional Review Board (No. 2213/2018).

### Data Collection and Follow-Up Data

Anamnestic, tumor and therapy data were collected and updated in the EUSOMA-accredited database, DataBreast. Each patient’s data are updated on a yearly basis until 5 years of follow-up are reached, at least. In order to identify the appropriate disease-free time, every type of relapse was reported with related date and localization.

### Evaluation of Inflammatory Biomarkers

For all patients, laboratory data on cell blood count was exported as electronic medical record from the hospital management system (clinical electronic repositories). Only preoperative blood counts, i.e. taken within 90 days before surgery, were considered for the analysis. For each patient, blood count closer to the date of surgery were selected. Of the 1,935 patients who met the inclusion criteria, 1,763 patients (91.0%) with available data for preoperative blood counts were included in the study. Hence, the following parameters were calculated: NLR, PLR and LMR.

### Study Design and Outcome Assessment

The primary endpoint of the study was to assess the prognostic role of preoperative NLR, PLR, LMR on 5-years locoregional recurrence-free survival (LRRFS), distant metastases-free survival (DMFS) and disease-free survival (DFS). First, we determined the optimal cutoff points to predict LRRFS, DMFS and DFS through time-dependent Receiver Operating Characteristic (ROC) analysis for NLR, PLR and LMR. Then these ratios were marked as “low” or “high” according to the above-mentioned cutoffs. LRR was defined as the occurrence of ipsilateral breast cancer and/or axillary relapse proven by biopsy. DM was defined as the evidence of distant lesions demonstrated by imaging (computed tomography and positron emission tomography) even if not histologically proven. Univariate and multivariate survival analyses for LRRFS, DMFS and DFS were performed, considering both blood inflammatory biomarkers and clinical-pathological variables.

### Statistical Analysis

Variables were reported as means and standard deviations with relative range or as absolute numbers and percentages. Categorical variables were compared using χ2 test, while continuous variables were compared using Student’s t-test or non-parametric Wilcoxon test in case of non-normal distribution of the variable. A Cox proportional hazard regression model was performed in order to identify possible effects of each variable significantly associated with the survival events in a time-dependent setting. Five-years survival probabilities were estimated by the Kaplan-Meier method both globally and in specific subsets. Statistical significance was set at p<0.05 (two tailed). Univariate and multivariate analyses were performed to assess the prognostic role of NLR, PLR, LMR on long-term patient outcome. Age at diagnosis, pathological assessment of the tumor (pT) and the regional lymph nodes (pN), Ki67, biological portrait, grade and histological type of the tumors were selected *a priori* as relevant clinical variables to be included in the multivariate analysis. A time-dependent ROC analysis was performed in order to identify the optimal cutoff values for each parameter. A Cohen’s kappa (K-Cohen) was assessed for agreement calculation between inflammatory biomarkers-based estimate of the risk for survival events and traditional clinical risk assessment by the modified version of Adjuvant!Online (20). Data analysis was performed using SAS software (v. 9.4, SAS Institute Inc., Cary, USA) and R software (v. 3.5.1, ^©^ The R Foundation).

## Results

### Characteristics of the Study Population

1,763 breast cancer patients were included in the study and 43 patients presented with bilateral lesions, for a total of 1,806 cancer cases examined. Demographics and clinical-pathological features of the cases included in the study are presented in **Table 1**. The mean age at diagnosis was 62 (± 13) years and 74.6% of the patients were postmenopausal. In 1,345 cases (74.5%) a conservative surgery was performed, while 461 breast lesions (25.5%) were treated by mastectomy. Ductal and lobular tumors represented respectively 78% and 15.4% of the cases. The majority of the cases were pT1 stage (81.3%), node negative (70.4%) tumors. Biomolecular subtype was ER+ HER2- in 80.9% of the cases, ER+ HER2+ in 8.5%, ER- HER2- in 6.5% and ER- HER2+ in 4.1%. Disease recurrence occurred in 91 cases (5.4%) as LRR and in 106 cases (5.9%) as DM. **Supplementary Figure 1** shows the Kaplan-Meier curves for 5-years DFS, LRRFS and DMFS of the study population.

**Table 1:** Clinical and pathological characteristics of the study population (n=1806 breast lesions).

Variable	BC (n = 1806)	Variable	BC (n = 1806)
**Age at diagnosis (years)**	62 ± 13 [26–95]	**pN**	
**BMI**	26.7 ± 25.5 [14.2-46.1]	0	1204 (70.2%)
**Hormonal Status**		1	360 (21%)
Fertile	439 (24.3%)	2	88 (5.1%)
Pregnancy	2 (0.1%)	3	63 (3.7%)
Menopause	1347 (74.6%)	**Biological portrait**	
Replacement therapy	18 (1%)	ER+/HER2-	1368 (80.9%)
**Type of surgery**		ER+/HER2+	144 (8.5%)
Conservative surgery	1345 (74.5%)	ER-/HER2+	69 (4.1%)
Mastectomy	461 (25.5%)	ER-/HER2-	110 (6.5%)
**Axillary dissection**		**PG**	
No	1180 (65.3%)	Negative	384 (21.3%)
Yes	626 (34.7%)	Positive	1422 (78.7%)
**LNS biopsy**		**Ki67**	
No	316 (17.5%)	≤ 14%	1177 (65.2%)
Yes	1490 (82.5%)	> 14%	629 (34.8%)
**Type of breast cancer**		**Radiotherapy**	
Microinvasive	27 (1.5%)	No	504 (28.1%)
Invasive	1779 (98.5%)	Yes	1288 (71.9%)
**Histological type**		**Chemotherapy**	
Ductal	1409 (78%)	No	1218 (68.2%)
Lobular	278 (15.4%)	Yes	567 (31.8%)
Others	119 (6.6%)	**Biological therapy**	
**Grading**		No	1487 (89.6%)
I	190 (10.6%)	Yes	173 (10.4%)
II	1133 (63.2%)	**Hormonal therapy**	
III	469 (26.2%)	No	307 (17.2%)
**Lymphovascular invasion**		Yes	1476 (82.8%)
No	1053 (58.6%)	**Exitus**	
Yes	744 (41.4%)	No	1779 (98.0%)
**Tumor dimension (mm)**	15 ± 9 [0-100]	Yes	37 (2.0%)
**pT**		**DM**	
X	7 (0.4%)	No	1700 (94.1%)
1	1468 (81.3%)	Yes	106 (5.9%)
2	305 (16.9%)	**Time to DM (months)**	25 ± 25 [0-60]
3	12 (0.6%)	**LRR**	
4	14 (0.8%)	No	1715 (92.6%)
		Yes	91 (7.4%)
		**Time to LRR (months)**	25 ± 25 [0-60]

BC, breast cancer; BMI, body mass index; ER, estrogen receptor; HER2, human epidermal growth factor receptor 2; PG, progesterone receptor; DM, distant metastasis; LRR, locoregional recurrence.

Data are expressed as mean ± standard deviation or total numbers; range and frequency distribution are shown within square and round parentheses, respectively.

### Survival Outcomes According to NLR, PLR, LMR

**Figure 1:**
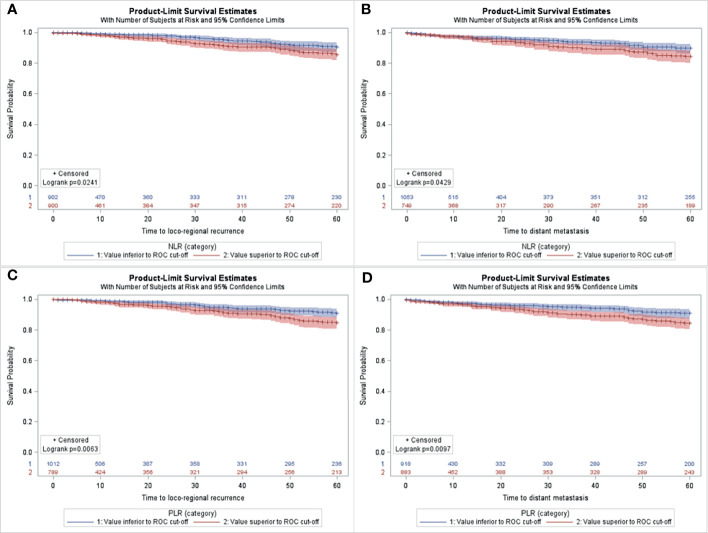
Kaplan-Meier curves for LRRFS **(A, C)** and DMFS **(B, D)** according to low *vs.* high NLR **(A, B)** or PLR **(C, D)** in the study population (n=1806).

**Figure 2:**
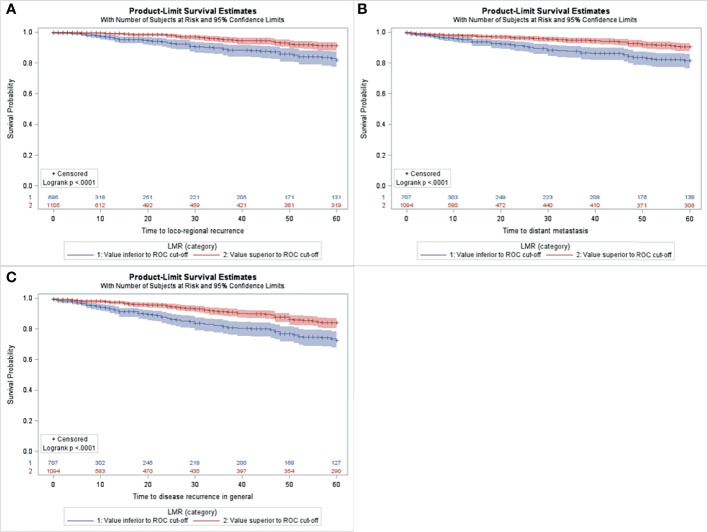
Kaplan-Meier curves for LRRFS **(A)**, DMFS **(B)**, DFS **(C)** according to low *vs.* high LMR in the study population (n=1806).

For the whole series, median preoperative NLR was 2.28 ± 1.25 (range 0.16-19.00), median PLR was 133.38 ± 51.9 (range 10.75-459.14) and median LMR 3.97 ± 1.52 (range 0.60-31.0). Based on the ROC analyses, the optimal cutoff values of NLR, PLR and LMR were calculated for each survival outcome (see **Supplementary Table 1**). Patients with a low NLR had a significantly longer 5-years LRRFS and DMFS than those with high NLR (**Figures 1A, B**). Similarly, the group with low PLR showed increased LRRFS and DMFS when compared to the group with high PLR (**Figures 1C, D**). Moreover, patients with high LMR displayed LRRFS, DMFS, and DFS longer than those with low LMR (**Figure 2**). No association between NLR or PLR and DFS was observed.

Multivariate Cox analysis showed that high preoperative NLR was an independent predictor of worse 5-years LRRFS (HR 0.51; p=0.005), DMFS (HR 0.65; p=0.04) and DFS (HR 0.68; p=0.02). In addition high baseline values of PLR had an independent significant impact on 5-years LRRFS (HR 0.56; p=0.01) and DMFS (HR 0.62; p=0.03). High LMR values were independently associated with improved 5-years LRRFS (HR 2.36; p=0.0003), DMFS (HR 2.06; p=0.0009) and DFS (HR 1.92; p=0.0001). Other than the inflammatory blood biomarkers herein described, age at diagnosis, pT and pN status, and tumor biological subtype, especially hormone receptor status, were found main independent risk factors for recurrence. Data obtained from the multivariate analysis are shown in **Table 2**; results from the univariate analysis are reported as **Supplementary Table 2**.

**Table 2:** Multivariate analysis of inflammatory and clinical characteristics in relation to 5-years LRRFS, DMFS, DFS in the study population (n=1806).

Variables	LRRFS			DMFS			DFS		
	HR	95%CI	p	HR	95%CI	p	HR	95%CI	p
**NLR**									
Low	0.51	0.32-0.82	0.005	0.65	0.43-0.98	0.04	0.68	0.49-0.94	0.02
High	Ref.			Ref.			Ref.		
**Age**	1.03	1.01-1.04	0.005	1.02	1.00-1.03	0.06	1.02	1.01-1.03	0.0009
**pT**									
pT1	0.72	0.42-1.24	0.24	0.48	0.31-0.76	0.002	0.60	0.42-0.88	0.008
pT2/3/4	Ref.			Ref.			Ref.		
**pN**									
pN0/1	0.97	0.52-1.79	0.92	0.31	0.20-0.48	<0.0001	0.52	0.35-0.75	0.0006
pN2/3	Ref.			Ref.			Ref.		
**Ki67**									
≤14%	0.66	0.39-1.12	0.12	0.79	0.49-1.28	0.34	0.70	0.48-1.03	0.07
>14%	Ref.			Ref.			Ref.		
**Biological portrait**									
ER+/HER2–	0.34	0.16-0.72	0.005	0.37	0.18-0.75	0.006	0.40	0.23-0.70	0.001
ER+/HER2+	0.29	0.09-0.91	0.03	0.59	0.26-1.35	0.21	0.49	0.24-1.00	0.05
ER-/HER2+	0.73	0.27-1.93	0.52	0.61	0.23-1.6	0.32	0.64	0.3-1.35	0.24
ER-/HER2-	Ref.			Ref.			Ref.		
**Grade**									
G1/2	1.22	0.64-2.32	0.55	0.90	0.53-1.51	0.68	1.03	0.67-1.59	0.89
G3	Ref.			Ref.			Ref.		
**Histological type**									
Lobular	0.98	0.51-1.85	0.94	1.14	0.66-1.96	0.64	1.06	0.68-1.64	0.81
Others	0.84	0.30-2.32	0.73	0.53	0.17-1.69	0.28	0.61	0.27-1.40	0.25
Ductal	Ref.			Ref.			Ref.		
**PLR**									
Low	0.56	0.35-0.88	0.01	0.62	0.40-0.96	0.03	0.75	0.54-1.05	0.09
High	Ref.			Ref.			Ref.		
**Age**	1.03	1.01-1.05	0.003	1.02	1.00-1.03	0.03	1.02	1.01-1.04	0.0005
**pT**									
pT1	0.71	0.41-1.22	0.22	0.46	0.29-0.72	0.0007	0.59	0.41-0.85	0.005
pT2/3/4	Ref.			Ref.			Ref.		
**pN**									
pN0/1	0.95	0.52-1.76	0.87	0.30	0.19-0.47	<0.0001	0.51	0.35-0.74	0.0004
pN2/3	Ref.			Ref.			Ref.		
**Ki67**									
≤14%	0.64	0.38-1.09	0.10	0.79	0.49-1.28	0.34	0.70	0.48-1.02	0.06
>14%	Ref.			Ref.			Ref.		
**Biological portrait**									
ER+/HER2–	0.35	0.16-0.74	0.006	0.37	0.18-0.75	0.005	0.40	0.23-0.7	0.001
ER+/HER2+	0.31	0.10-0.96	0.04	0.58	0.25-1.32	0.19	0.49	0.24-1.00	0.05
ER-/HER2+	0.69	0.26-1.82	0.45	0.62	0.24-1.61	0.32	0.63	0.30-1.33	0.22
ER-/HER2-	Ref.			Ref.			Ref.		
**Grade**									
G1/2	1.22	0.64-2.32	0.55	0.94	0.56-1.59	0.82	1.05	0.68-1.62	0.83
G3	Ref.			Ref.			Ref.		
**Histological type**									
Lobular	0.95	0.50-1.80	0.86	1.12	0.66-1.93	0.67	1.05	0.68-1.63	0.83
Others	0.85	0.31-2.36	0.75	0.57	0.18-1.83	0.34	0.63	0.28-1.44	0.27
Ductal	Ref.			Ref.			Ref.		
**LMR**									
Low	2.36	1.49-3.75	0.0003	2.06	1.35-3.16	0.0009	1.92	1.37-2.68	0.0001
High	Ref.			Ref.			Ref.		
**Age**	1.02	1.01-1.04	0.01	1.01	1.00-1.03	0.10	1.02	1.01-1.03	0.002
**pT**									
pT1	0.72	0.42-1.23	0.23	0.48	0.30-0.75	0.001	0.59	0.41-0.86	0.006
pT2/3/4	Ref.			Ref.			Ref.		
**pN**									
pN0/1	0.93	0.50-1.72	0.82	0.29	0.19-0.45	<0.0001	0.49	0.33-0.72	0.0002
pN2/3	Ref.			Ref.			Ref.		
**Ki67**									
≤14%	0.61	0.36-1.04	0.07	0.75	0.47-1.21	0.24	0.68	0.47-0.99	0.05
>14%	Ref.			Ref.			Ref.		
**Biological portrait**									
ER+/HER2–	0.29	0.14-0.62	0.001	0.33	0.16-0.67	0.002	0.36	0.20-0.62	0.0003
ER+/HER2+	0.26	0.08-0.83	0.02	0.53	0.23-1.21	0.13	0.44	0.21-0.90	0.03
ER-/HER2+	0.63	0.24-1.66	0.35	0.59	0.23-1.55	0.29	0.61	0.29-1.29	0.19
ER-/HER2-	Ref.			Ref.			Ref.		
**Grade**									
G1/2	1.32	0.69-2.51	0.40	0.96	0.57-1.62	0.88	1.09	0.71-1.69	0.69
G3	Ref.			Ref.			Ref.		
**Histological type**									
Lobular	1.00	0.53-1.90	1.00	1.15	0.67-1.98	0.61	1.08	0.69-1.68	0.73
Others	0.88	0.32-2.43	0.80	0.56	0.18-1.81	0.34	0.66	0.29-1.51	0.32
Ductal	Ref.			Ref.			Ref.		

### Performance of Survival Prediction by Inflammatory Blood Biomarkers *vs.* Clinical-Pathological Features

In order to better understand if NLR, PLR, LMR provided different and innovative information than clinical-pathological features, agreement calculation with Cohen’s kappa was assessed between clinical risk assessment and preoperative inflammatory blood biomarkers. The two different approaches were not in agreement for every biomarker (**Supplementary Table 3**), suggesting that the prognostic value of NLR, PLR and LMR on survival events is not covered by the current clinical assessment of the disease.

### The Prognostic Role of NLR, PLR, LMR in ER+ HER2- Early Breast Cancer

From the whole patient dataset, 1,547 early breast lesions were selected and defined as pT1-2 and pN0-1 tumors. Baseline features and cutoff values of NLR, PLR, LMR in this subset were calculated and reported as **Supplementary Tables 4**, **5**. By multivariate analysis we found that preoperative NLR, PLR and LMR were independent prognostic factors for LRRFS in early breast cancer (HR 0.57; p=0.03, HR 0.55; p=0.02, HR 1.86; p=0.02, respectively) (**Supplementary Table 6**).

We then focused on 999 ER+ HER2- early breast cancers, which were treated by hormonotherapy without chemotherapy. For these patients, timely risk stratification is important in order to escalate or de-escalate appropriate adjuvant therapy. Optimal cutoff values of preoperative NLR, PLR LMR for the prediction of LRRFS, DMFS, DFS in this patient population were re-calculated (**Table 3**). The multivariate analysis showed that high PLR was significantly predictive of worse 5-years LRRFS (HR 0.42, p=0.009), while high LMR was predictive of improved 5-years LRRFS (HR 2.20, p=0.02) and DFS (HR 2.10, p=0.01), as reported in **Table 4**. Conversely, NLR was not an independent factor of 5-years survival in this group of patients. Other independent variables for LRRFS were age at diagnosis and Ki67 (only in the evaluation of DFS for LMR).

**Table 3:** Optimal cutoff values of preoperative NLR, PLR, LMR for prediction of 5-years LRRFS, DMFS, DFS in ER+ HER2- early breast cancers.

	LRRFS			DMFS			DFS		
	NLR	PLR	LMR	NLR	PLR	LMR	NLR	PLR	LMR
**AUC**	0.52	0.55	0.55	0.54	0.51	0.55	0.51	0.51	0.54
**Cutoff**	2.01	136.64	3.75	2.22	119.80	3.61	2.01	119.87	3.75

**Table 4:** Multivariate analysis of inflammatory and clinical characteristics in relation to 5-years LRRFS, DFS in ER+ HER2- early breast cancers not treated with chemotherapy (n=999).

Variables	LRRFS			Variables	LRRFS			DFS		
	HR	95%CI	p		HR	95%CI	p	HR	95%CI	p
**PLR**				**LMR**						
Low	0.42	0.22-0.81	0.009	Low	2.20	1.11-4.37	0.02	2.10	1.17-3.74	0.01
High	Ref.			High	Ref.			Ref.		
**Age**	1.03	1.01-1.06	0.01	**Age**	1.03	1.00-1.06	0.03	1.04	1.01-1.06	0.002
**pT**				**pT**						
pT1	1.16	0.38-3.57	0.80	pT1	1.14	0.38-3.48	0.81	0.82	0.37-1.79	0.61
pT2	Ref.			pT2	Ref.			Ref.		
**pN**				**pN**						
pN0	0.76	0.33-1.75	0.52	pN0	0.77	0.34-1.77	0.54	0.62	0.33-1.18	0.14
pN1	Ref.			pN1	Ref.			Ref.		
**Ki67**				**Ki67**						
≤14%	0.49	0.23-1.05	0.07	≤14%	0.58	0.27-1.23	0.16	0.48	0.26-0.88	0.01
>14%	Ref.			>14%	Ref.			Ref.		
**Grade**				**Grade**						
G1/2	1.13	0.36-3.55	0.83	G1/2	1.02	0.32-3.21	0.98	0.90	0.39-2.07	0.80
G3	Ref.			G3	Ref.			Ref.		
**Histological type**				**Histological type**						
Lobular	0.60	0.23-1.58	0.30	Lobular	0.65	0.25-1.71	0.38	0.61	0.27-1.38	0.24
Others	1.10	0.33-3.72	0.87	Others	1.14	0.34-3.83	0.83	0.78	0.24-2.56	0.68
Ductal	Ref.			Ductal	Ref.			Ref.		

## Discussion

This study shows that systemic lymphocyte ratios, as measured in preoperative blood samples, can be reliable and inexpensive markers of disease recurrence in an unselected cohort of breast cancer patients. In particular, patients with high NLR and PLR had a significantly shorter 5-years LRRFS and DMFS, while the ones with high LMR had longer survival outcomes. As expected, multivariate analysis associated other factors to poor prognosis: age at diagnosis, pT and pN status, and ER status. More importantly, as for ER+ HER2- early breast cancers not treated with chemotherapy, PLR and LMR were found to be independent predictors of 5-years LRRFS, and LMR predicted both LRRFS and DFS.

Lymphocyte ratios have drawn an increasing attention in different fields of medicine, as they can be easily assessable markers of inflammation and prognosis in several disorders. From a pathophysiological point of view, a state of systemic inflammation is associated to an increased tumor aggressiveness due to the pro-angiogenic oxidative state that favours the acquisition of a stem cell status as well as the impairment of DNA repair mechanisms. Multiple studies have shown that higher NLR is associated with poorer survival in metastatic breast cancer (21, 22) and a recent meta-analysis highlighted that higher NLR was associated with both worse DFS and overall survival (12). Several previous studies reported that higher NLR is also associated with more advanced and aggressive breast cancer (23, 24). For this reason, the ratios between neutrophils in blood and other leukocytes, as the NLR, have been suggested as a prognostic value in cancer (25, 26). NLR is higher in patients with a more advanced disease (24), and correlates with poor survival in many cancers (27). However, recent studies showed controversial evidences of NLR usefulness in hormone receptor-positive breast cancer (28, 29). NLR, simple and inexpensive biomarker, has been introduced as a significant prognostic factor in many tumor types (30). However, it has not been accepted in many clinical settings since neutrophilia can be the result of elevated granulopoiesis and, therefore, may not be considered as an adverse sign for cancer progression. Another reason is that neutrophilia is associated with poor clinical outcome in all cancers except for stomach cancer, in which case a high NLR is a marker of good prognosis (27).

In this study, we analyzed simultaneously NLR, PLR and LMR as potential inflammatory biomarkers, and all of them showed concordant prognostic results in terms of 5-years LRRFS in early breast cancer patients. Interestingly we did not observe any overlap between clinical risk assessment and NLR, PLR, LMR in the prediction of survival outcomes. This suggests that the information derived from inflammatory biomarkers is different and non-redundant with the clinical features of the tumor currently available. Indeed, preoperative lymphocyte ratios may be more related to the patient’s immune system rather than being associated with the tumor burden, especially in case of early breast cancers. Therefore, easy-gettable lymphocyte ratios from routine blood counts may provide precious prognostic data to be added to the standard clinical assessment of the tumor.

Regarding ER+ HER2- early breast cancer, we found that preoperative PLR and LMR are prognostic biomarkers of disease recurrence. This piece of data may be helpful in clinics, where failure of standard therapy (endocrine treatment and chemotherapy) is observed in a substantial portion of ER+ HER2- early breast cancers (31–33). Therefore, non-invasive, inexpensive and easy obtained circulating biomarkers may contribute to select those patients who will benefit from personalized and scaled-up adjuvant treatments.

The main strengths of this study are: the large cohort of patients presented, the data homogeneously collected, and the simultaneous assessment of different lymphocyte ratios. However there are some limitations and pitfalls worth mentioning. First, the time span of the registry is rather long, so cancer therapy, types, and prognosis might have changed over time. Secondly, our findings may have been biased by the retrospective nature of the study. Lastly, we did not evaluate the stromal TILs in the tumors. Literature data demonstrate a robust association between stromal TILs and better prognoses, in particular in triple negative and HER2+ breast cancers (34–36). In these breast cancer subtypes high levels of TILs are also associated with increased response to neoadjuvant and adjuvant chemotherapy (37–39). However, a defined prognostic and predictive role of TILs in luminal-like breast cancer is still debated, likely due to the biological heterogeneity of this breast cancer subtype (40, 41). As future perspective, further studies will be undertaken to quantify TILs in selected cohorts of early breast cancers, with the aim to correlate the systemic inflammatory biomarkers with the corresponding picture of the immune infiltrate in the tissue.

In conclusion, our data suggest that preoperative systemic inflammatory blood biomarkers could provide clinically relevant information regarding the risk of disease relapse in early breast cancer, especially in case of ER+ HER2- tumors generally considered as good prognosis. Further studies assessing the clinical suitability of these markers are required. Moreover, postoperative inflammatory biomarkers should also deserve attention to determine the course of some treatments, by assessing the changes occurring during treatments in appropriately designed prospective studies.

## Data Availability Statement

The original contributions presented in the study are included in the article/**Supplementary Material**. Further inquiries can be directed to the corresponding author.

## Ethics Statement

The studies involving human participants were reviewed and approved by the Institutional Review Board of Istituti Clinici Scientifici Maugeri (No. 2213/2018). The patients/participants provided their written informed consent to participate in this study.

## Author Contributions

MT and FC contributed to conception and design of the study. MT, FP, SA, and VT organized the database and collected data. SA and CM analyzed the data. MT, SA, FS, and FC drafted the manuscript. All authors contributed to manuscript revision, read, and approved the submitted version.

## Funding

Article processing fee was paid by Istituti Clinici Scientifici Maugeri IRCCS.

## Conflict of Interest

The authors declare that the research was conducted in the absence of any commercial or financial relationships that could be construed as a potential conflict of interest.

## Publisher’s Note

All claims expressed in this article are solely those of the authors and do not necessarily represent those of their affiliated organizations, or those of the publisher, the editors and the reviewers. Any product that may be evaluated in this article, or claim that may be made by its manufacturer, is not guaranteed or endorsed by the publisher.
