# Supplementary material for: Preoperative Systemic Inflammatory Biomarkers Are Independent Predictors of Disease Recurrence in ER+ HER2- Early Breast Cancer
Source: Front Oncol. 2021 Nov 4;11:773078. doi: 10.3389/fonc.2021.773078 (PMC8600180; doi:10.3389/fonc.2021.773078)
Supplement: Supplementary file 1 [file DataSheet_1.pdf]

Supplementary Material

Supplementary Figure 1. Kaplan-Meier curves for 5-years LRRFS (A), DMFS (B), DFS (C) in the study population (n=1806)

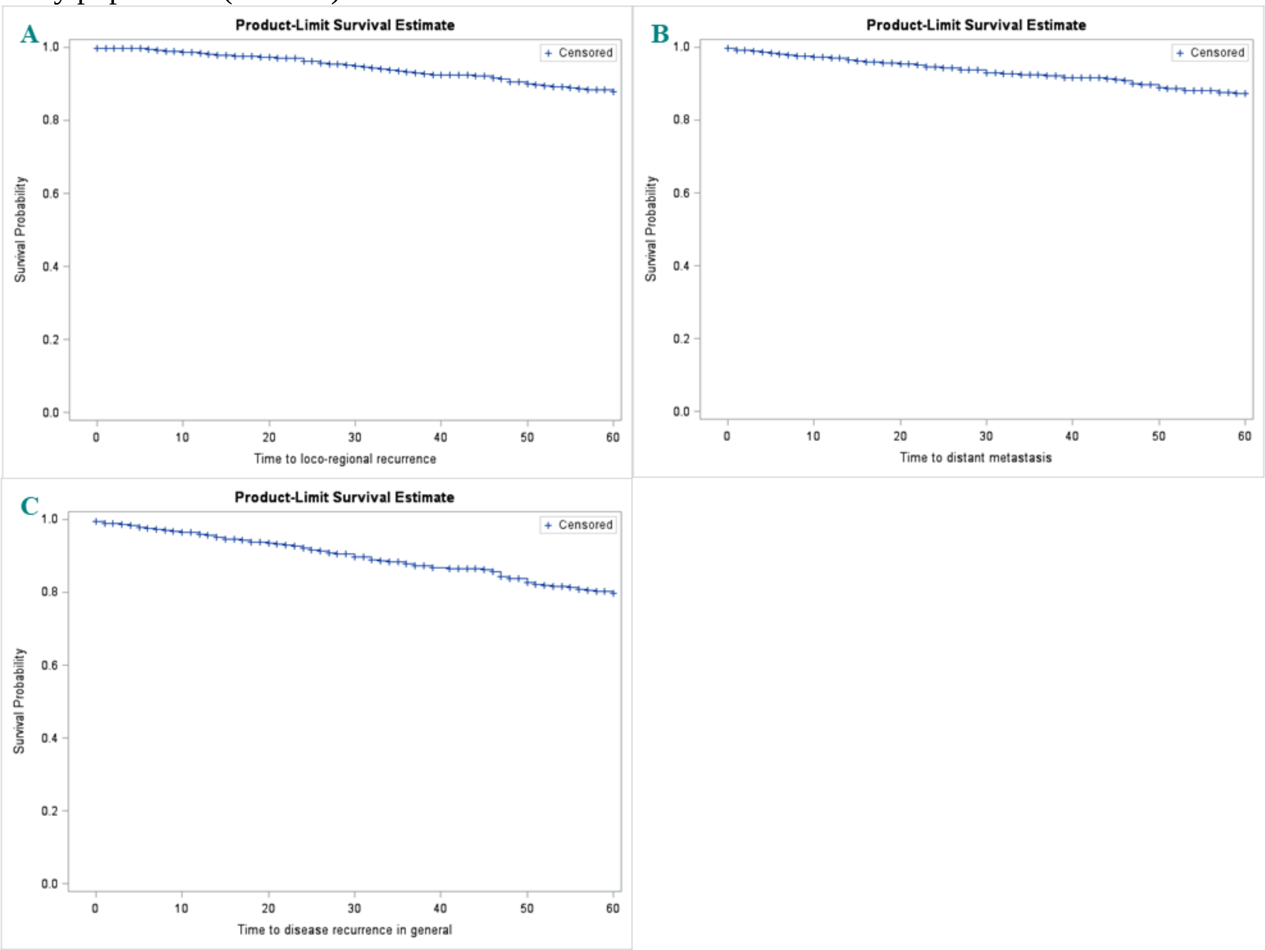

Supplementary Table 1. Optimal cutoff values of pre-operative NLR, PLR, LMR for prediction of 5-years LRRFS, DMFS, DFS in the study population (n=1806)

	LRR			DM			DF		
	**NLR**	**PLR**	**LMR**	**NLR**	**PLR**	**LMR**	**NLR**	**PLR**	**LMR**
**AUC**	0.56	0.59	0.61	0.58	0.56	0.60	0.54	0.54	0.59
**Cutoff**	2.01	130.5	3.48	2.21	124.1	3.49	2.21	126.8	3.49

Supplementary Table 2. Hazard ratios (HR) and 95% confidential intervals (CI) for 5-years LRRFS, DMFS, DFS in low vs. high NLR, PLR and LMR groups (n=1806)

	LRRFS			DMFS			DFS		
	**HR**	**95%CI**	**p-value**	**HR**	**95%CI**	**p-value**	**HR**	**95%CI**	**p-value**
**NLR**	1.62	1.06-2.49	0.03	1.48	1.01-2.17	0.04	1.6	1.00-1.85	0.05
**PLR**	1.79	1.17-2.74	0.007	1.69	1.13-2.52	0.01	1.35	0.99-1.84	0.06
**LMR**	0.43	0.29-0.65	<0.0001	0.47	0.32-0.69	0.0001	0.51	0.38-0.69	<0.0001

Supplementary Table 3. Agreement calculation (K-Cohen) between clinical risk assessment and NLR, PLR, LMR

		LRR			DM			DF		
		CRS		K-Cohen	CRS		K-Cohen	CRS		K-Cohen
		Low	High		Low	High		Low	High	
**NLR**										
	Low	445	378	0.008	521	437	0.02	521	437	0.02
	High	421	370		345	311		345	311	
**PLR**										
	Low	509	424	0.02	463	385	0.02	483	399	0.02
	High	357	323		403	362		383	348	
**LMR**										
	Low	313	292	-0.03	321	295	-0.02	321	295	-0.02
	High	553	456		545	453		545	453	

Supplementary Table 4. Clinical and pathological characteristics of early breast cancer population (n=1547). Data are expressed as mean ± standard deviation or total numbers; range and frequency distribution are shown within square and round parentheses, respectively

**Variable**	**Early BC (n=1547)**	**Variable**	**Early BC (n=1547)**
**Age at diagnosis (years)**	61±13 [26-90]	**pN**	
**BMI**	24.9±4,9 [14.2-46.1]	0	1197 (77.4%)
**Hormonal Status**		1	350 (22.6%)
Fertile	390 (25.2%)	**Biological portrait**	
Pregnancy	1 (0.1%)	ER+/HER2–	1185 (81.5%)
Menopause	1141 (73.7%)	ER+/HER2+	122 (8.4%)
Replacement therapy	15 (1.0%)	ER-/HER2+	50 (3.4%)
**Type of surgery**		ER-/HER2-	97 (6.7%)
Conservative surgery	1200 (77.6%)	**PG**	
Mastectomy	347 (22.4%)	Negative	322 (20.8%)
**Axillary dissection**		Positive	1225 (79.2%)
No	1088 (70.3%)	**Ki67**	
Yes	459 (29.7%)	≤ 14%	1035 (66.9%)
**LNS biopsy**		> 14%	512 (33.1%)
No	136 (8.8%)	**Radiotherapy**	
Yes	1411 (91.2%)	No	418 (27.2%)

**Type of breast cancer**		Yes	1118 (72.8%)
Microinvasive	23 (1.5%)	**Chemotherapy**	
Invasive	1524 (98.5%)	No	1111 (72.7%)
**Histological type**		Yes	418 (27.3%)
Ductal	1226 (79.3%)	**Biological therapy**	
Lobular	225 (14.5%)	No	1288 (89.7%)
Others	96 (6.2%)	Yes	148 (10.3%)
**Grading**		**Hormonal therapy**	
I	176 (11.4%)	No	233 (15.3%)
II	999 (64.9%)	Yes	1295 (84.8%)
III	364 (23.7%)	**Exitus**	
**Lymphovascular invasion**		No	1526 (98.6%)
No	959 (62.3%)	Yes	21 (1.4%)
Yes	581 (37.7%)	**DM**	
**Tumor dimension (mm)**	14±7 [0-50]	No	1481 (95.7%)
**pT**		Yes	66 (4.3%)
1	1322 (85.5%)	**Time to DM (months)**	24±25 [0-60]
2	225 (14.5%)	**LRR**	
		No	1473 (95.2%)
		Yes	74 (4.8%)
		**Time to LRR (months)**	25±25 [0-60]

Supplementary Table 5. Optimal cutoff values of preoperative NLR, PLR, LMR for prediction of 5-years LRRFS, DMFS, DFS in early breast cancers (n=1547)

	**LRRFS**			**DMFS**			**DFS**		
	**NLR**	**PLR**	**LMR**	**NLR**	**PLR**	**LMR**	**NLR**	**PLR**	**LMR**
**AUC**	0.54	0.57	0.57	0.55	0.53	0.57	0.51	0.51	0.55
**Cutoff**	2.01	145.88	3.75	2.18	132.95	3.61	2.08	130.99	3.75

Supplementary Table 6. Multivariate analysis of inflammatory and clinical characteristics in relation to 5-years LRRFS, DMFS, DFS in early breast cancers (n=1547)

Variables	LRRFS			DMFS			DFS		
	HR	95%CI	p	HR	95%CI	p	HR	95%CI	p
**NLR**									
Low	0.57	0.34-0.95	0.03	0.67	0.39-1.16	0.15	0.77	0.52-1.13	0.18
High	Ref.			Ref.			Ref.		
**Age**	1.03	1.01-1.05	0.01	1.02	1.00-1.05	0.02	1.03	1.01-1.04	0.001
**pT**									
pT1	0.83	0.42-1.65	0.60	0.53	0.29-0.97	0.04	0.67	0.41-1.09	0.10
pT2	Ref.			Ref.			Ref.		
**pN**									
pN0	0.96	0.54-1.72	0.89	0.44	0.25-0.76	0.004	0.69	0.45-1.06	0.09
pN1	Ref.			Ref.			Ref.		
**Ki67**									
≤14%	0.59	0.32-1.07	0.08	0.48	0.25-0.91	0.02	0.48	0.30-0.75	0.001
>14%	Ref.			Ref.			Ref.		
**Biological portrait**									
ER+/HER2–	0.34	0.15-0.80	0.01	0.41	0.18-0.95	0.04	0.41	0.22-0.77	0.005
ER+/HER2+	0.29	0.08-1.07	0.06	0.72	0.25-2.05	0.54	0.44	0.18-1.09	0.08
ER-/HER2+	0.60	0.19-1.9	0.38	0.31	0.07-1.41	0.13	0.49	0.19-1.25	0.14
ER-/HER2-	Ref.			Ref.			Ref.		
**Grade**									
G1/2	1.35	0.62-2.94	0.45	1.08	0.53-2.21	0.83	1.28	0.74-2.24	0.38
G3	Ref.			Ref.			Ref.		
**Histological type**									
Lobular	1.06	0.52-2.13	0.88	1.10	0.51-2.4	0.80	1.03	0.59-1.78	0.93
Others	1.10	0.39-3.12	0.85	0.38	0.05-2.82	0.35	0.67	0.24-1.83	0.43
Ductal	Ref.			Ref.			Ref.		
**PLR**									
Low	0.55	0.33-0.91	0.02	0.85	0.49-1.46	0.55	0.85	0.58-1.26	0.42
High	Ref.			Ref.			Ref.		
**Age**	1.03	1.01-1.05	0.01	1.03	1.00-1.05	0.02	1.03	1.01-1.04	0.001
**pT**									
pT1	0.82	0.41-1.63	0.57	0.50	0.27-0.92	0.03	0.66	0.41-1.07	0.09
pT2	Ref.			Ref.			Ref.		
**pN**									
pN0	1.01	0.56-1.80	0.99	0.44	0.25-0.78	0.005	0.71	0.46-1.08	0.11
pN1	Ref.			Ref.			Ref.		
**Ki67**									
≤14%	0.55	0.30-1.01	0.06	0.47	0.25-0.88	0.02	0.47	0.30-0.75	0.001
>14%	Ref.			Ref.			Ref.		
**Biological portrait**									
ER+/HER2–	0.35	0.15-0.81	0.01	0.43	0.19-0.98	0.05	0.42	0.22-0.78	0.006
ER+/HER2+	0.31	0.08-1.14	0.08	0.74	0.26-2.09	0.57	0.46	0.19-1.13	0.09
ER-/HER2+	0.58	0.18-1.85	0.36	0.30	0.06-1.37	0.12	0.48	0.19-1.22	0.12

ER-/HER2-	Ref.			Ref.			Ref.		
**Grade**									
G1/2	1.40	0.64-3.04	0.40	1.09	0.53-2.23	0.82	1.28	0.73-2.22	0.39
G3	Ref.			Ref.			Ref.		
**Histological type**									
Lobular	1.03	0.51-2.07	0.94	1.09	0.50-2.37	0.82	1.03	0.59-1.78	0.92
Others	1.13	0.40-3.19	0.82	0.37	0.05-2.75	0.33	0.66	0.24-1.83	0.43
Ductal	Ref.			Ref.			Ref.		
**LMR**									
Low	1.86	1.12-3.09	0.02	1.39	0.81-2.39	0.23	1.38	0.94-2.03	0.11
High	Ref.			Ref.			Ref.		
**Age**	1.03	1.01-1.05	0.02	1.02	1.00-1.05	0.03	1.03	1.01-1.04	0.001
**pT**									
pT1	0.77	0.39-1.53	0.46	0.50	0.27-0.92	0.03	0.65	0.40-1.05	0.08
pT2	Ref.			Ref.			Ref.		
**pN**									
pN0	1.00	0.56-1.80	1.00	0.45	0.25-0.78	0.005	0.71	0.46-1.08	0.11
pN1	Ref.			Ref.			Ref.		
**Ki67**									
≤14%	0.59	0.33-1.08	0.09	0.48	0.25-0.91	0.02	0.48	0.31-0.76	0.002
>14%	Ref.			Ref.			Ref.		
**Biological portrait**									
ER+/HER2–	0.33	0.14-0.77	0.01	0.41	0.18-0.95	0.04	0.40	0.21-0.75	0.004
ER+/HER2+	0.31	0.08-1.14	0.08	0.72	0.25-2.05	0.54	0.46	0.19-1.12	0.09
ER-/HER2+	0.53	0.17-1.68	0.28	0.29	0.06-1.34	0.11	0.47	0.19-1.19	0.11
ER-/HER2-	Ref.			Ref.			Ref.		
**Grade**									
G1/2	1.35	0.62-2.92	0.45	1.08	0.53-2.21	0.83	1.29	0.74-2.25	0.38
G3	Ref.			Ref.			Ref.		
**Histological type**									
Lobular	1.06	0.53-2.14	0.87	1.11	0.51-2.41	0.79	1.03	0.60-1.79	0.90
Others	1.15	0.41-3.27	0.79	0.39	0.05-2.85	0.35	0.69	0.25-1.89	0.47
Ductal	Ref.			Ref.			Ref.		
